# Competitive males have higher quality sperm in a monogamous social bee

**DOI:** 10.1186/s12862-016-0765-2

**Published:** 2016-09-27

**Authors:** Sheina Koffler, Hiara Marques Meneses, Astrid de Matos Peixoto Kleinert, Rodolfo Jaffé

**Affiliations:** 1 Department of Ecology, University of São Paulo, Institute of Biosciences, Rua do Matão, travessa 14, 321 (05508-090) São Paulo, SP Brazil; 2 Department of Animal Sciences, University of Ceará, Center of Agrary Sciences, Fortaleza, CE Brazil; 3 Vale Institute of Technology, Sustainable Development, Belém, PA Brazil

**Keywords:** Aggregation, Male competition, Sexual selection, Sperm, Social insects

## Abstract

**Background:**

Reproductive success is determined by the interplay between mating and fertilization success. In social insect species with male-biased sex ratios and queen monogamy, males face particularly strong pre-copulatory sexual selection since they must compete with thousands of other males for a unique mating opportunity. Ejaculate quality is also expected to be under selection, because queens are long-lived and store sperm for life, so males with higher quality ejaculates are expected to provide queens with larger and longer-lived colonies, which in turn may produce more daughter queens (the only direct fitness gains of haplodiploid males). Considering the action of pre and post-copulatory sexual selection on male traits, three scenarios might thus be expected: positive, negative or no association between male mating ability and fertilization success. Here we explored these scenarios in the stingless bee *Scaptotrigona* aff. *depilis*, where males gather in large aggregations and queens mate with a single male. Male mating ability was assessed through the capacity of a male to reach an aggregation and persist on it; while sperm viability, sperm number, and sperm morphology were used as proxies for sperm quality.

**Results:**

Sperm viability was associated with persistence time in the aggregation, and males that persisted longer presented shorter spermatozoa and higher variation in sperm length than recently arrived males. However, sperm traits of males that reached aggregations did not differ from those of males collected inside their colonies. In addition, males that persisted longer in aggregations were smaller than other males. Male size and sperm viability were not correlated, suggesting that the observed patterns were not due to trade-offs in male resource allocation.

**Conclusions:**

Persistence in male aggregations thus seems to select more competitive males with higher quality sperm. Our work is the first one to reveal an association between male competitive ability and fertilization success in a monogamous social insect. This finding sheds important light on the evolution of male traits in social insects and the general mechanisms of sexual selection.

**Electronic supplementary material:**

The online version of this article (doi:10.1186/s12862-016-0765-2) contains supplementary material, which is available to authorized users.

## Background

Reproductive success is determined by the interplay between individual mating and fertilization success. While mating success is subject to pre-copulatory sexual selection, via male-male competition and female choice [1, 2], fertilization success is usually related to post-copulatory sexual selection in species with multiple mating, through sperm competition [3] and cryptic female choice [4]. Considering these distinct selective episodes on males, attempts have been made to integrate pre and post-copulatory sexual selection, by understanding how males allocate limited resources into different traits [5, 6] and what is the genetic basis of these trade-offs [7, 8]. Pre- and post-copulatory sexual selection may exhibit synergistic or opposite effects, as pre-copulatory sexual selection outcomes may be reinforced by post-copulatory sexual selection [9, 10], or in other cases, post-copulatory sexual selection may attenuate pre-copulatory sexual selection [8, 11–13].

In monogamous species, selection for fertilization ability is expected to be more relaxed than in polygamous species, since the main selective pressure is to achieve copulation and insemination [14, 15]. Here, male resource allocation should have been selected to invest into traits maximizing the chances of insemination, as sperm entering the female reproductive tract will not compete with sperm from other males. However, in monogamous species where egg fertilization does not occur immediately after mating, male fertilization ability may also be subject to selection. In these species, females normally possess sperm-storage organs, where sperm are kept viable until fertilization [16]. Both mating and fertilization abilities are thus expected to be under selection in males of monogamous species with sperm-storage, making them particularly well suited to study post-copulatory sexual selection in the absence of sperm competition.

The social Hymenoptera (ants, bees and wasps) exhibit one of the longest sperm-storage periods in the animal kingdom, with queens mating once in life and storing sperm for several years, during which they continuously lay eggs [17–19]. In this group of haplodiploid insects, males are haploid because they originate from unfertilized eggs, whereas fertilized eggs give rise to diploid females. Since reproductive success is determined by the production of sexual offspring (males and daughter queens, also known as gynes), males only achieve direct fitness gains through the production of gynes [20]. As sexual offspring are only produced in strong and well established colonies, most sperm is used to produce sterile workers [21, 22]. Male reproductive success is thus directly dependent on their fertilization capacity, which will translate into workers and later in gynes, so stored sperm is expected to be under selection for quantity and quality.

Stingless bees are an ideal model system to study the interplay between mating and fertilization success in the absence of sperm competition, given that they have extremely male-biased sex ratios (strong selection for mating success), and monandrous long-lived queens (strong selection for fertilization success) [18, 23–27]. During reproductive events, hundreds to thousands of males gather in aggregations waiting for an opportunity to mate with a virgin queen [28, 29]. This mating system results in intense male competition for mating, as males must find an aggregation, persist on it until a queen arrives, chase her on the flight, and finally achieve copulation and insemination. Whether the queen chooses a mate [30] remains unknown, but the extreme male-biased sex ratios imply that male competition is the predominant form of pre-copulatory sexual selection [2, 18]. On the other hand, ejaculate quality is expected to be under selection, since queens live up to several years, mate only once in life, and maintain colonies that can harbor thousands of individuals [27, 31, 32]. Considering that selection is acting both before and after mating, three scenarios might be expected: 1) male competitive and fertilization abilities are positively related, with competitive males also exhibiting high ejaculate quality; 2) male competitive and fertilization abilities are negatively related, with males exhibiting higher competitive ability or higher ejaculate quality, but not both; and 3) male competitive and fertilization abilities are uncorrelated.

**Fig. 1:**
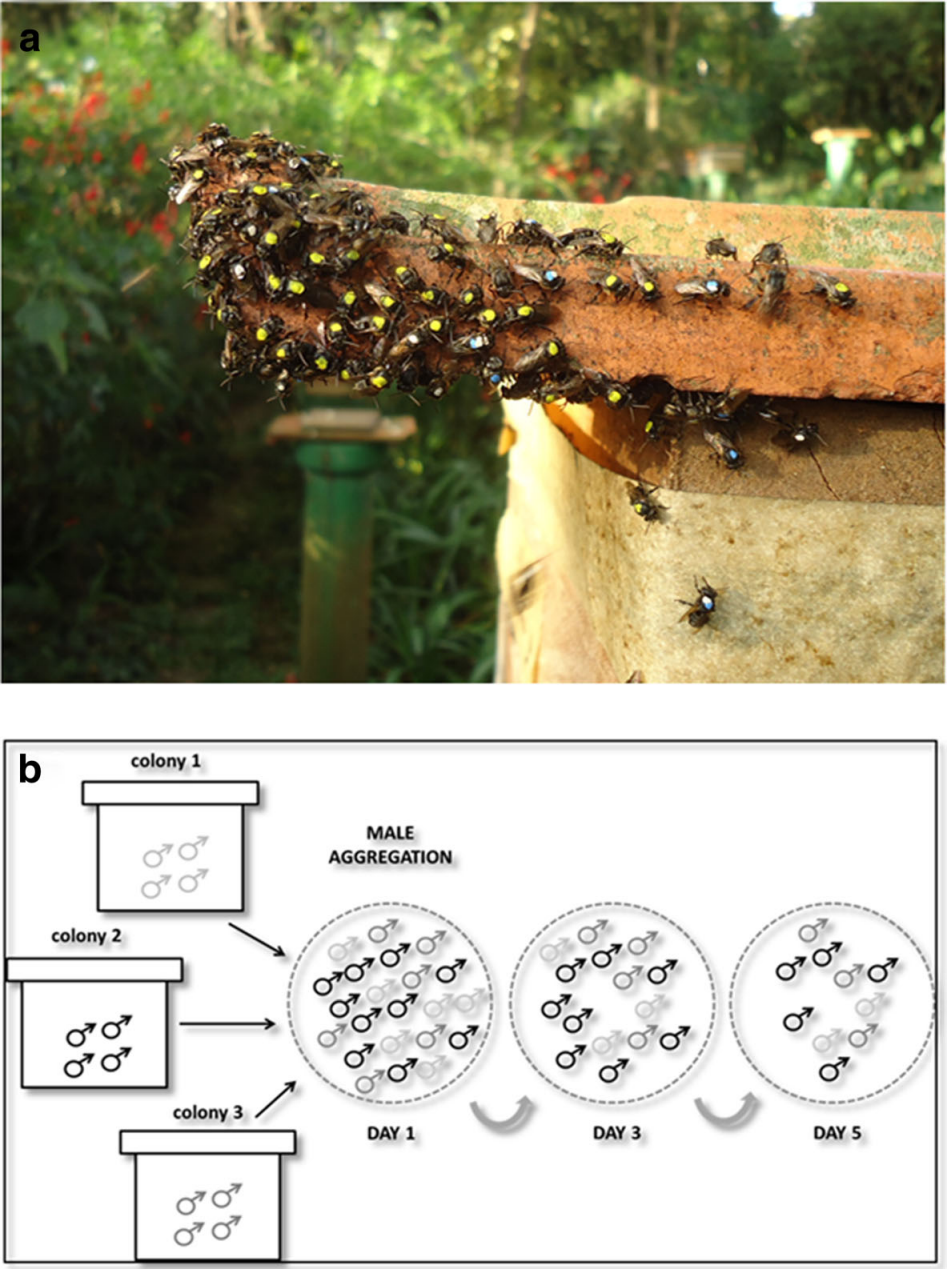
**a**. Male aggregation of *Scaptotrigona* aff. *depilis*, above a colony of the same species. The individuals are paint-marked in the thorax. **b** Representation of the sampling designs performed to discriminate males with different competitive ability. In the first selection episode investigated, the capacity of males to reach an aggregation was analyzed (black arrows) and males collected inside the colonies were compared to males collected at aggregations. In the second selection episode, male persistence in the aggregations was analyzed (gray arrows), and new-coming males and males that persisted for three and 5 days were compared

Here we investigated the relationship between male competitive ability and fertilization success in the stingless bee *Scaptotrigona* aff. *depilis*, a species exhibiting large male aggregations [33] that are found throughout the year (Fig. 1a). Two contrasting hypotheses were explored: 1) male traits exposed to pre- and post-copulatory sexual selection are related (either positively, which predicts that more competitive males would exhibit higher quality sperm, or negatively, which predicts that competitive males would exhibit lower quality sperm, and males exhibiting high quality sperm would be less competitive); 2) pre-copulatory traits are unrelated to post-copulatory traits. To examine these hypotheses, we first identified a set of behavioral traits that successfully predicted male competitive ability (ability to reach and persist in an aggregation). We then employed a model-selection protocol to relate these traits to different indicators of ejaculate quality (sperm viability, sperm counts, and sperm length), thus effectively integrating mating and fertilization success. We also identified morphological traits under pre-copulatory sexual selection affecting male competitive ability and investigated potential trade-offs in male resource allocation into different traits. We discuss the implications of our findings for the evolution of male traits in social insects, the operation of sexual selection in this group, and the general theory on integrating pre and post-copulatory sexual selection.

## Methods

Mating flights are rarely observed in stingless bees, making it difficult or impossible to compare males that mated successfully with males that failed to mate. Moreover, after mating, males lose their genitalia and die [18]. To account for these particular features, male competitive ability was analyzed at two selection episodes before mating: 1) leaving the colony and reaching an aggregation, and 2) persisting in the aggregation (Fig. 1b). In the first selection episode, males collected inside colonies were compared to males from the same colonies that successfully reached an aggregation. In the second selection episode, the number of days spent at the aggregation was measured.

Morphological and sperm quality indicators were analyzed in all males. Since chemical and visual cues play important roles in the aggregation process [34], we expected that competitive males would present better sensory structures (larger eyes and longer antennae). In addition, as males showed conspicuous directional asymmetry in the compound eyes, we tested if the degree of asymmetry was associated with male performance. We also expected that competitive males would present larger body size, as larger sizes are related to higher mating success in other social insects [35–37]. To assess male fertilization ability, sperm viability, number, length, and length variation were measured. Males with higher fertilization ability were expected to exhibit higher sperm viability, which allows efficient sperm storage and fertilization [38–40]. Sperm number is also expected to be related to fertilization success, given that sufficient sperm are needed for long term fertilizations [19, 41]. Since producing short sperm cells is less costly and allows the storage of higher amounts of sperm in the queen’s spermathecae [18], we expected sperm length to be negatively associated to fertilization ability. Finally, as selection is expected to reduce sperm length around an optimal length [42], males with higher fertilization ability are also expected to exhibit lower sperm length variation.

### Assessing mating success 1: from the colony to the aggregation

Five colonies of *Scaptotrigona* aff. *depilis* earlier inspected for male production were selected and kept in laboratory conditions (University of São Paulo, São Paulo, SP, Brazil). Each colony was maintained indoors, in a wooden box covered with glass lids and connected to the outside by a plastic tube, so the bees had free access to the environment. All brood combs with pre-emergent pupae were collected from the colonies and kept in individually labeled Petri dishes placed inside an incubator (28 ± 1 °C), with sugar water solution. Emerging males were paint-marked on the thorax (using UniPosca® pens) every second day, for four continuous days. Males from each colony were identified by the same color and were returned to the colony after marking. Since males were previously found to reach aggregations between 17 to 20 days after emergence (in our study location, Additional file 1: Figure S1), between 2 and 31 males were collected from each colony 15 days after marking (Table 1). These hive-collected males were kept in Petri dishes in the incubator and provided with sugar water solution ad libitum. Aggregations formed in the vicinity of the colonies were then daily inspected for marked males, which were collected for two continuous weeks. Four aggregations were found in the outside area of the laboratory and males were collected from all aggregations, to maximize sample size. Aggregation-collected males were kept in the incubator, following the same procedure described above, until sperm viability analyses were performed. In order to avoid great age differences among males from the two sampling groups (hive and aggregation), which could affect sperm viability measures (Additional file 2: Figure S2), males collected from the aggregations were analyzed together with the same number of hive-collected males. Males from three colonies were included in the analyses, since only two males of the remaining colonies were collected (Table 1).

**Table 1:** Comparing males collected inside colonies with males collected at aggregations

Colony	Number of marked males	Number of marked males collected inside the colonies	Number of marked males collected at aggregations	Proportion of marked males collected at aggregations (%)
1	507	31	51	10
2	461	30	32	7
3	362	30	24	6
4	110	26	2	2
5	77	2	1	1

Assessing mating success 2: persistence in aggregations

Data are presented for number of marked males, number of marked males collected inside the colonies 15 days after marking, and number and proportion of males collected at aggregations during the 2 weeks of inspection

We then quantified persistence time in the aggregations, using different groups of males (all the previously marked males had died by the time we began assessing persistence time). We first selected a large aggregation and proceeded to mark all males on their thorax with the same color. The marking process continued throughout a full day, until only marked males were found in the aggregation. On the following day, new-coming males (with no paint marks) were marked with a different color, following the same procedure. This second group of males, which had a known arrival date, was then subsequently collected in the aggregation during the following 5 days. Males were collected haphazardly, using a net, and collection was always performed at the same interval time (1 to 3 pm) in the defined days. Previous observations revealed that males show high fidelity to the visited aggregation, as less than 1 % of the marked males were found in different aggregations. We followed this protocol to quantify persistence time in May 2014 (*n* = 233 marked males) and again in May 2015 (*n* = 68 marked males). In 2015, only small aggregations were found and males from two aggregations were followed (each aggregation marked with a different color, *n* = 30 and *n* = 38). Males were collected in the marking day (new-coming males – persistence of 1 day), after 2 days in the aggregation (males that persistence for 3 days), and after 4 days in the aggregation (males that persisted for 5 days). All males collected from the aggregations were kept inside an incubator (28 ± 1 °C) in Petri dishes with sugar water solution ad libitum for 1 day, until dissection for sperm viability analyses were performed. Assays were previously performed in order to test whether male age affected sperm viability, as found for honey bee drones [40]. Emerging males from two colonies were collected and kept in individual wooden boxes, inside an incubator, and receiving sugar syrup and pollen ad libitum. Weekly, 10 males were sampled and sperm viability was analyzed, repeating this procedure for 5 weeks. As sperm viability was found to decrease with male age (Additional file 2: Figure S2), and males were at least 17 days old when they arrived at the aggregation, we did not collect males that persisted more than 5 days in the aggregations (around 3 weeks old).

### Measurement of male traits

To assess morphological traits (Additional file 3: Table S1), males were kept frozen after dissections (see below). Male head and thorax were photographed using a stereomicroscope coupled with a camera (20x magnification) and measurements were made using the open source software ImageJ [43]. Male heads were placed over foam sheet material and covered with a glass lid. We used intertegular distance (the shortest distance between the bases of the tegulae) as an estimate of bee size [44], and also quantified maximum head length, total eye area (area of the left and right compound eyes in frontal view) and antennae length. Eye asymmetry was calculated as the difference between the left eye area and right eye area.

To assess ejaculate quality we measured sperm viability (relative proportion of live sperm cells), total sperm cell number and sperm length, following standard protocols [45]. Male’s abdomens were pressed until the exposure of the endophallus and the two seminal vesicles, which were then removed with forceps and placed in 120 μl of Hayes solution (9 g of NaCl, 2 g of CaCl_2_, 0.2 g of KCl, and 0.1 g NaHCO_3_, to 1 l of deionized water, pH adjusted to 8.7). Vesicles were ruptured and emptied using a pin and the semen solution was gently homogenized with a pipette. We excluded samples if the male died before dissection or the vesicles ruptured inside the male’s abdomen. To perform sperm viability analysis, 5 μl of the semen solution was stained with 5 μl of SYBR14 working solution (2 μl of SYBR14 in 98 μl of Hayes solution) and 2 μl of propidium iodide (LIVE/DEAD ® Sperm Viability Kit). Microscope slides were inspected in a fluorescence microscope at 40x magnification and 400 cells in each sample were analyzed, counting live (green), dead (red) and dying (both colors) spermatozoa. Double stained cells represented less than 5 % of counts in all samples and were considered as live spermatozoa [19]. When comparing sperm viability of hive-collected and aggregation-collected males, we only included data from the first week of sampling, because hive-collected males that remained in the incubator for two weeks were not comparable to freshly collected males from the aggregations. Sperm length was assessed by spreading 10 μl of sperm solution in a microscope slide. Each sample was air dried and stained with 30 μl of DAPI (4 ng/μl to 100 ng/μl) and photographs were taken at 20x magnification in a fluorescence microscope. For each male, 10 sperm cells were measured using the software ImageJ, and mean sperm length (from the tip of the head to the end of the tail) and the coefficient of variation (ratio of standard deviation to the mean) were calculated. Sperm counts were only performed for males collected in 2015, and were assessed by diluting 12 μl of the semen solution in 988 μl of Hayes solution. Five samples of 1 μl of the diluted sperm were added to a microscope slide. Each sample was air-dried and stained with 5 μl DAPI (4 ng/μl) and all sperm heads from three samples from each male were counted at 40x magnification. Sperm counts were then multiplied by the dilution factor (x 10,000).

### Statistical analyses

In order to identify which morphological traits were exposed to pre-copulatory sexual selection, male competitive ability was modeled in relation to the different morphological traits assessed. Since some morphological traits (head width, intertegular distance, eye area and left antennae length) were positively correlated, these variables were merged using Principal Components Analysis. The first component (PC1) explained 59.8 % of the variation in male morphology in the sample of hive-collected and aggregation-collected males (scores were positively correlated to the size of morphological traits), and 75.6 % of the variation in the sample of males with different persistence times (scores were negatively correlated with the size of morphological traits, and were thus multiplied by −1). The probability of reaching the aggregation (presence/absence in the aggregation) was analyzed fitting a generalized linear mixed effects model with binomial distribution, including a random effect for the colony of origin and an observation-level random effect to account for overdispersion. Colony of origin was not included as a fixed factor in our models, because we were not interested in the particular effects of colony on male performance. Persistence time (number of days in the aggregation) was modeled fitting a generalized linear mixed effects model with Poisson distribution, with a random factor for aggregation identity. In both analyses, male size (PC1) and eye asymmetry were included as predictors in a full model. Predictors were scaled, by centering the mean on zero and dividing by the standard deviation, to aid model convergence. We used Likelihood Ratio Tests (LRT, alpha = 0.05) to reduce the number of predictor to those significantly improving our model’s log-likelihood. We compared full models with models where each predictor was excluded, and selected those which significantly (LRT *p* < 0.05) increased the model’s log-likelihood (as described in [46]). We thus assessed the individual effect of all predictors accounting for other covariates, and tested all possible combination of predictors. Overdispersion was diagnosed by calculating the sum of squared Pearson residuals and comparing it to the residual degrees of freedom (using a *χ*2 distribution to estimate the *p*-value) and by plotting residuals vs. fitted values (sperm length data). The significance of estimates in the final model was also tested using a Wald test.

Models relating male fertilization ability and male competitive ability were constructed to assess the relationship between traits exposed to pre- and post-copulatory sexual selection. We used the ability to reach and persist in an aggregation as a direct measure of male quality, given that the morphological traits influencing male competitive ability exhibited higher variation and only represent indirect measures (see below). Each sperm trait was modeled using linear mixed effects models, with the sperm trait as response variable and male competitive ability (sampling site or sampling time) as predictor (see Table 2 for details). Sperm viability was modeled as the proportion of live sperm cells, using a binomial distribution. When comparing males from the hives versus males from the aggregations, the number of days in the incubator was also included as fixed factor (interacting with sampling site), in order to account for a possible influence of the incubator on sperm viability. Sperm number was modeled as the number of sperm cells in each of the three samples from each male, using a Poisson distribution and a random effect for replicate. Sperm length and sperm length variation were modeled using a linear mixed model. Random effects were included to account for the dependence among samples (samples from the same colony, aggregation or male) or to account for overdispersion when necessary (observation-level random effect). Model selection was performed using Likelihood Ratio Tests as described above.

**Table 2:** Models relating male sperm quality to male competitive ability

Selection episode	Model	Response	Probability distribution	Predictors	Random effect
Colony -Aggregation	sperm viability	proportion of live to dead sperm cells	Binomial	sampling site and number of days in incubator	colony and male^a^
	sperm length	sperm length	Normal	sampling site	colony and male
	sperm length variation	sperm CV	Normal	sampling site	colony
Persistence time in the aggregation	sperm viability	proportion of live to dead sperm cells	Binomial	days at aggregation	aggregation and male^a^
	sperm number	number of sperm cells	Poisson	days at aggregation	aggregation and male
	sperm length	sperm length	Normal	days at aggregation	aggregation and male
	sperm length variation	sperm CV	Normal	days at aggregation	aggregation

Each trait was analyzed according to a particular probability distribution and random effects were included to account for dependence among samples or overdispersion

^a^Observation level random effect to account for overdispersion

Finally, we correlated the morphological and sperm measures selected in the best models in the previous analyses, to check for trade-offs in resource allocation, using Spearman’s correlation. All analyses were performed in R, using the *lme4* [48] and *Hmisc* [49] packages. Plots were produced with the package *ggplot2* [47].

## Results

During 2 weeks, about 5 % of all marked males reached four different aggregations outdoors (Table 1). The number of marked males in each colony was positively correlated to the number of marked males collected at the aggregations (r_s_ = 0.96, *p* = 0.008, *n* = 5).

**Fig. 2:**
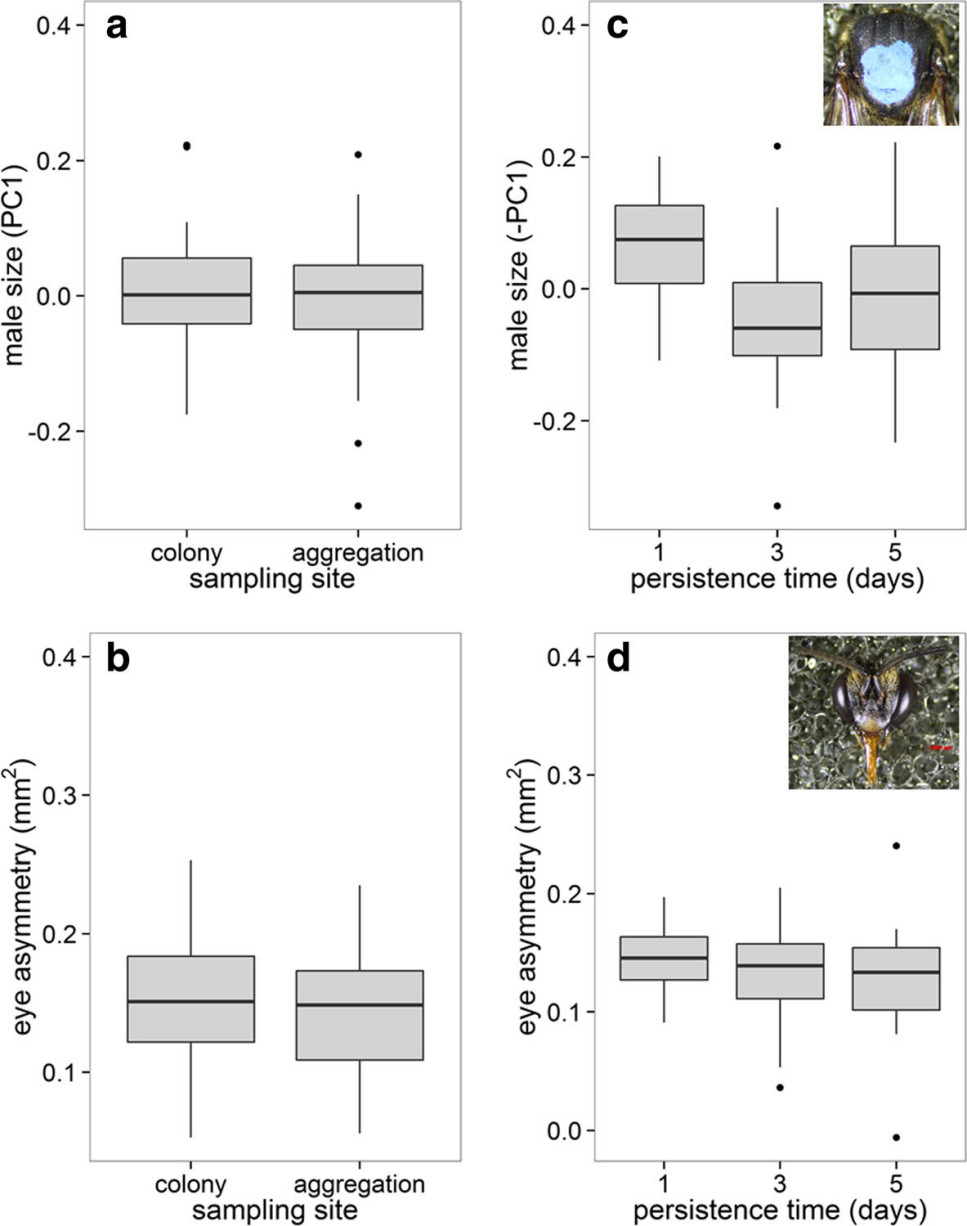
Morphological traits related to male competitive ability (male’s ability to reach and persist in aggregations). Neither male body size nor eye asymmetry influenced the male’s ability to reach an aggregation (**a**-**b**). Males that persisted more days in the aggregations were smaller, but did not show different eye asymmetry (**c**-**d**). Median values are represented by the lines inside the boxes, which span the first and third quartiles, and points represent data outside 1.5 times the interquartile range above the upper quartile and bellow the lower quartile. Data from different colonies and aggregations were merged (see colony and aggregation effects in Additional file 4: Figure S3)

The probability of reaching an aggregation was not influenced by any male morphological trait, while the number of days that a male persisted at the aggregation was best predicted by male size (Table 3). Males that persisted longer at aggregations were usually smaller (Table 4, Fig. 2). Even though directional asymmetry was found in the male’s compound eyes (Fig. 2), with left eyes being always larger than right eyes (except for one male), the degree of eye asymmetry did not affect male competitive ability.

**Table 3:** Model selection table for models relating competitive ability and male morphology

Selection episode	N.obs	Response	Random effect	Starting model	Fixed effect removed	Degrees of freedom	*χ*2	*P*-value
Colony -Aggregation	110	Probability of reaching an aggregation	colony and male^a^	male size + eye	male size	1	0.65	0.42
				asymmetry	eye asymmetry	1	0.05	0.82
				male size	male size	1	0.67	0.41
				asymmetry	asymmetry	1	0.07	0.79
Persistence time in the aggregations	65	Number of days at the aggregation	aggregation	male size + eye	male size	1	4.56	0.03
				asymmetry	eye asymmetry	1	0.76	0.38
				male size	male size	1	5.17	0.02
				eye asymmetry	eye asymmetry	1	1.38	0.24

The *p*-values and degrees of freedom refer to Likelihood Ratio Tests (using a *χ*2 test statistic), in which the full model was compared to a reduced model without each of the predictor variables. Parameters estimates of best models are presented in Table 4

^a^Observation level random effect to account for overdispersion

**Table 4:** Best-fitting model for the relationship between competitive ability and male morphology

Selection episode	Response	Predictor	Parameter estimate	SE	*P*-value
Persistence time in the aggregations	Number of days at the aggregation	male size	−1.48	0.65	0.02

Males that persisted longer at the aggregation were smaller. Parameter estimates, standard errors and *p*-values are given

**Fig. 3:**
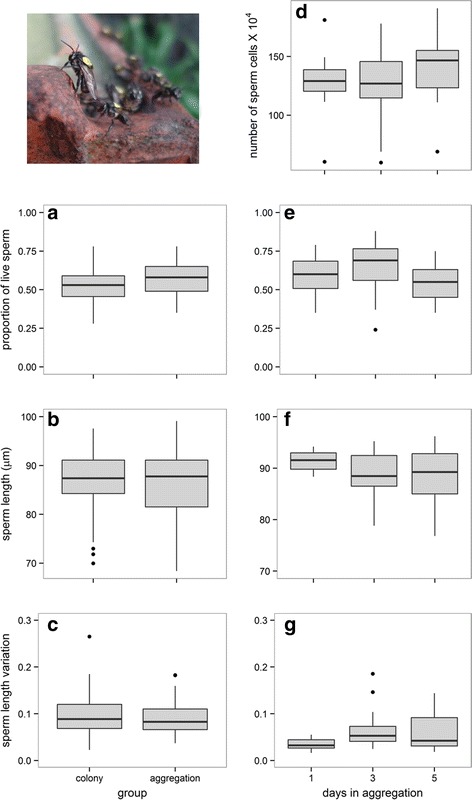
*Scaptotrigona* aff. *depilis* male at an aggregation. **a**-**c** Sperm traits of males collected inside the colonies and males that reached aggregations. **d**-**g** Sperm traits of males with different persistence times in the aggregations (new-coming males and males that persisted for 3 or 5 days). Median values are represented by the lines inside the boxes, which span the first and third quartiles, and points represent data outside 1.5 times the interquartile range above the upper quartile and bellow the lower quartile. Data from different colonies and aggregations were merged (see colony and aggregation effects in Additional file 5: Figure S4)

No sperm trait affected the probability of a male reaching an aggregation, as in our comparisons of hive and aggregation-collected males the best-fitting models were the intercept-only models (Tables 5 and 6, Fig 3a-c). The time spent in the incubator did not affect sperm viability. On the other hand, sperm viability, length and length variation were associated with persistence time in the aggregations (Table 5, Fig. 3d-g). Males that persisted longer at the aggregations exhibited shorter spermatozoa and higher sperm length variation. However, the differences in sperm viability between days were not significant (Table 6). Male persistence time in the aggregations was not associated with sperm number.

**Table 5:** Model selection table for different models testing the relationship between male competitive ability and fertilization success (sperm traits)

Selection episode	N. obs	Response	Starting model	Fixed effect removed	Degrees of freedom	*χ*2	*P*-value
Colony - Aggregation	47	Sperm viability	sampling site*days in incubator	interaction	1	0.05	0.82
			sampling site + days in incubator	sampling site	1	0.29	0.59
			sampling site + days in incubator	days in incubator	1	0.004	0.95
			sampling site	sampling site	1	1.82	0.18
			days in incubator	days in incubator	1	1.54	0.21
	1070	Sperm length	sampling site	sampling site	1	0.01	0.91
	107	Sperm length variation	sampling site	sampling site	1	0.12	0.73
Persistence time in the aggregation	60	Sperm viability	days at aggregation	days at aggregation	2	7.28	0.03
	90	Sperm number	days at aggregation	days at aggregation	2	3.77	0.15
	609	Sperm length	days at aggregation	days at aggregation	2	12.70	<0.01
	61	Sperm length variation	days at aggregation	days at aggregation	2	16.07	<0.001

The *p*-values and degrees of freedom refer to Likelihood Ratio Tests (using a *χ*2 test statistic), in which the full model was compared to a reduced model without each of the predictor variables. Parameters estimates of best models are presented in Table 5

**Table 6:** Best-fitting models describing the relationship between male competitive ability and fertilization success (sperm traits)

Selection episode	Response	Predictor	Parameter estimate		SE	*P*-value
Persistence time in the aggregation	Sperm viability	Days at aggregation	3 days	0.26	0.18	0.15
			5 days	−0.24	0.18	0.18
	Sperm length	Days at aggregation	3 days	−3.36	0.98	<0.01
			5 days	−3.02	0.99	<0.01
	Sperm length variation	Days at aggregation	3 days	0.03	0.08	<0.001
			5 days	0.03	0.08	<0.01

For each predictor, parameter estimates, standard errors and *p*-values are given

Correlation between male size (which affected competitive ability) and sperm traits revealed that body size was not significantly correlated to sperm viability (r_s_ = 0.06, *p* = 0.49, *n* = 148), but was marginally correlated to mean sperm length (r_s_ = 0.16, *p* = 0.053, *n* = 153) and negatively correlated to sperm length variation (r_s_ = −0.17, *p* = 0.04, *n* = 153).

## Discussion

Our results reveal that male competitive ability in *Scaptotrigona* aff. *depilis* is associated with male size, but not by eye asymmetry. Sperm traits from males that reached aggregations did not differ from sperm traits of hive-collected males, whereas male persistence time in the aggregations was associated with variation in sperm viability, sperm length and sperm length variation. Males that persisted longer in the aggregations usually showed shorter sperm cells and higher sperm length variation. Overall, our findings suggest that aggregations select high quality males, as male competitive ability was positively related to ejaculate quality.

### Identifying morphological traits under pre-copulatory sexual selection

Colonies that produced more males also presented a higher number of males in the aggregations, as found in honey bee drone [35]. This finding suggests that colony fitness may be enhanced by the number of males produced. However, male quality could also influence colony fitness by affecting male performance in the aggregations. Indeed, competitive males were found to be smaller, contradicting the initial predictions and previous studies with social insects, which found a positive association between male size and mating success [35–37]. This finding could be related to a trade-off between investment in body size and sperm traits, although no correlations between the assessed traits were found (see below). In swarming species, even though larger males show greater longevity, smaller males have higher mating success [50], which is attributed to agility in flight while in the swarm [51]. Male contests, on the other hand, usually select for larger body size [18]. In our case, as fights do not occur among males in aggregations of *S.* aff. *depilis* (personal observation), investing in larger body sizes would not provide a competition advantage. In contrast, in *Melipona favosa* aggressive behaviors have been observed between males in the aggregation [28]. In addition, larger males may incur in higher energy consumption, which may hinder long distance flights during the search for an aggregation, or compromise the ability to persist longer in an aggregation [52]. Male size may also be subject to other forms of natural selection, as longevity, predator avoidance [53] or immune defense.

Most males exhibited some degree of directional asymmetry in the compound eyes (the left eyes always larger than the right eyes). Even though directional asymmetries can be related to male quality signals, as in birds [54] and crickets [55], the degree of eye asymmetry did not influence male competitive ability in our study. More studies are thus needed to understand the possible adaptive function of the marked eye asymmetry in stingless bee males and if the larger left eye may act as a compensation for a differential visual performance.

### Relationship between male competitive and fertilization abilities

Even though males spent up to 5 days in the incubator, sperm viability did not vary with the number of days males were kept in the incubator. This indicates that short term male confinement is feasible for sperm viability analysis, which greatly facilitates experimental designs with large sample sizes. Sperm viability did not vary between hive-collected males and males that reached aggregations, but it was associated to male persistence time at the aggregation (including sperm viability as a predictor that significantly improved our model’s log-likelihood, Table 5). Even though the effect sperm viability was weak (Table 6), males that persisted for 3 days tended to show higher sperm viability than new-coming males, while males that persisted for 5 days tended to show lower sperm viability. Future studies are thus needed to explore this effect in more detail, and understand why sperm viability increases at 3 days in the aggregation in spite of the negative effect of aging. Although a virgin queen mating with males that stay five or more days in the aggregation would lower her fertilization success, it remains unclear if the mating ability of males also changes through time, perhaps decreasing the chances of an older male mating with a queen.

On the other hand, sperm number did not affect male persistence time at the aggregation. Observed sperm number was in agreement with previous sperm count estimates in *Scaptotrigona* queen spermathecae [56], suggesting that all sperm is transferred from the male to the queen during mating. This result suggests that sperm traits other than sperm number may influence male fertilization ability. Sperm viability for instance may be more relevant for fertilization potential, as sperm cells must be live to provide successful egg fertilization and higher amounts of sperm may compromise viability.

Sperm length was negatively related to male persistence time in the aggregation. The production of short sperm cells is expected to be less costly [57], which could allow the production of a higher number of sperm cells or the reduction of the resources allocated to sperm production. Also, shorter cells enable a higher amount of sperm to be stored in queen’s spermathecae, resulting in higher long term fertilization potential if spermathecae volume is constrained [18].

Even though males that persisted longer in the aggregations presented shorter sperm cells, sperm length was also more variable, contradicting our initial expectations. This suggests no selective pressure acting to reduce sperm length variation, as described in polyandrous social species [42]. Previous work with *Bombus terrestris* revealed that sperm stored in queen’s spermathecae was less variable than sperm from male ejaculates, suggesting that during the storing process variance is removed by selecting a certain sperm length [58]. Hence, active (queen controlled) or passive (sperm related) processes occurring after mating may reduce variance in sperm length.

### Resource allocation trade-offs and concluding remarks

The fact that smaller males with higher sperm quality were selected in the aggregations suggests a trade-off between resource allocation in size and sperm quality, as found in leafcutter ant males [59]. However, we did not find a significant negative correlation between male size, sperm viability or sperm length. Our results thus fail to support trade-offs in resource allocation among these traits and suggest that two distinct selection episodes occur in male aggregations: one selecting smaller males, and the other selecting males with higher quality sperm.

Although no morphological or ejaculate difference was found between hive-collected and aggregation-collected males, we highlight that the studied colonies were very close to the aggregations (less than 30 m), which may have influenced our assays. On the other hand, male persistence in aggregations seems to select more competitive and fertile males. From the males that reach an aggregation, only the fittest ones are able to find shelter at night, avoid predators, cope with depleting energy reserves, and then return to the aggregation on the following days. Our results thus suggest that selective pressures acting on male aggregations may shape the evolution of male traits [18, 35, 60, 61]. As competitive males exhibited higher ejaculate quality, male aggregations may act as an indirect form of mate selection for queens, enhancing male competition through male persistence [62]. Similar processes were described for male swarms in paper wasps, in which endurance competition acts through male display duration [60]. This may be advantageous for the female when there is low opportunity for direct mate choice [62]. However, the mechanisms underlying direct female choice remain to be investigated [30].

Our results bring evidence of sexual selection acting on stingless bee males and reveal that competitive males exhibit higher fertilization potential, corroborating the hypothesis that male competitive and fertilization abilities are directly related. Male competition and long term sperm storage thus seem strong selective pressures shaping male traits in stingless bees. More generally, our findings reveal that ejaculate traits can be under post-copulatory sexual selection even in the absence of sperm competition. In contrast to our results, most studies relating male traits under pre- and post-copulatory sexual selection reveal trade-offs [13], although positive correlations have also been found [9, 10]. Models describing the evolution of sexual traits present two distinct scenarios according to the different mechanisms of male-male competition. Where contest competition occurs, males usually exhibit high investment in pre-copulatory sexual traits (weapons or ornaments), resulting in high female monopolization, and trade-offs between pre- and post-copulatory sexual traits are expected [63, 64]. On the other hand, in cases of scramble competition in polygynous species, the probability of female monopolization is low, and positive covariation is expected between pre and post-copulatory sexual traits [63]. Stingless bee mating system is in agreement with the second type, as it exhibits scramble competition features [18] and a positive relation between mating and fertilization abilities, but under monogamy. Even though males do not remate in this group, the mating system is marked by mate search and low opportunity for mate monopolization. Our findings are thus consistent with the theoretical expectations of scramble competition models in polygynous species and highlight that mechanisms other than sperm competition, such as long term sperm storage, may lead to a positive association between competition and fertilization ability.

## Conclusions

Stingless bee male aggregations seem to select more competitive males with higher quality sperm. Strong male competition and long term sperm storage are likely selective pressures shaping male traits in this group, showing that sperm quality may be under selection even in the absence of sperm competition. Our work is the first one to reveal an association between male competitive ability and fertilization success in a monogamous social insect. This finding sheds important light on the evolution of male traits in social insects and the general mechanisms of sexual selection.

## Additional files

Additional file 1: Figure S1. Arrival of mature *Scaptotrigona* aff. *depilis* males in aggregations, according to male age (days after emergence). Emerging males from three colonies were paint-marked in the thorax (each colony identified with a different color) and returned to their origin colonies (number of marked males: blue 206, beige 162, and green 60). Male aggregations were then inspected daily for marked males, which were counted when observed. (DOCX 12 kb) Additional file 2: Figure S2. Aging effect on male sperm viability (proportion of live to dead sperm cells), for males of two different colonies (each color represents a colony). Between nine and ten males per colony were analyzed weekly, from 1 week old to 5 weeks old. Median values are represented by the lines inside the boxes, which span the first and third quartiles, and points represent outliers. (TIF 91 kb) Additional file 3: Table S1. Male traits assessed in this study. (TIF 185 kb) Additional file 4: Figure S3. Morphological traits related to male competitive ability (male’s ability to reach and persist in aggregations). Neither male body size nor eye asymmetry influenced the male’s ability to reach an aggregation (a-b, each color represents a colony). Males that persisted more days in the aggregations were smaller, but did not show different eye asymmetry (c-d). Each color represents an aggregation (one aggregation was used in 2014 and two aggregations were used in 2015). Median values are represented by the lines inside the boxes, which span the first and third quartiles, and points represent data outside 1.5 times the interquartile range above the upper quartile and bellow the lower quartile. (TIF 326 kb) Additional file 5
**Figure S4**. (a-c) Sperm traits of males collected inside the colonies and males that reached aggregations. Each color represents a colony. (d-g) Sperm traits of males with different persistence times in the aggregations (new-coming males and males that persisted for 3 or 5 days). Each color represents an aggregation (one aggregation was used in 2014 and two aggregations were used in 2015). Median values are represented by the lines inside the boxes, which span the first and third quartiles, and points represent data outside 1.5 times the interquartile range above the upper quartile and bellow the lower quartile. (TIF 712 kb)
