# Supplementary material for: Competitive males have higher quality sperm in a monogamous social bee
Source: BMC Evol Biol. 2016 Sep 27;16:195. doi: 10.1186/s12862-016-0765-2 (PMC5039913; doi:10.1186/s12862-016-0765-2)
Supplement: Additional file 1: Figure S1. — Arrival of mature Scaptotrigona aff. depilis males in aggregations, according to male age (days after emergence). Emerging males from three colonies were paint-marked in the thorax (each colony identified with a different color) and returned to their origin colonies (number of marked males: blue 206, beige 162, and green 60). Male aggregations were then inspected daily for marked males, which were counted when observed. (DOCX 12 kb) [file 12862_2016_765_MOESM1_ESM.docx]

**Table S1.** Male traits assessed in this study. Morphological traits were measured to identify predictors of male competitive ability and sperm quality traits were measured to test the relationship between male fertilization success and competitive ability.

| **Trait type** | **Variable** | **Definition** |
| --- | --- | --- |
| Behavioral traits (male competitive ability) | success in reaching an aggregation | marked males collected in an aggregation and collected inside the colonies (binomial variable) |
|  | persistence time in the aggregation | number of days a male persisted in the aggregation (categorical variable: new-coming males, males persisting three and five days in the aggregation) |
| Morphological traits | intertegular span | shortest distante between the bases of the tegulae |
|  | head width | maximum head length (frontal view) |
|  | eye area | area of the left and right eyes (frontal view) |
|  | antennae length | length of the left and right antennae |
|  | eye asymmetry | difference between left and right eye area |
|  | male size (PC1) | first PCA score (PCA included intertegular span, head width, eye area and left antennae length) |
| Sperm quality traits | sperm viability | proportion of live sperm cells |
|  | sperm number | estimated number of sperm cells in the seminal vesicles |
|  | sperm length | length of sperm cell, from head to tail |
|  | sperm length variation | coefficient of variation of sperm cells length (sd/mean) |
