# Supplementary figures and images for: Competitive males have higher quality sperm in a monogamous social bee
Source: BMC Evol Biol. 2016 Sep 27;16:195. doi: 10.1186/s12862-016-0765-2 (PMC5039913; doi:10.1186/s12862-016-0765-2)

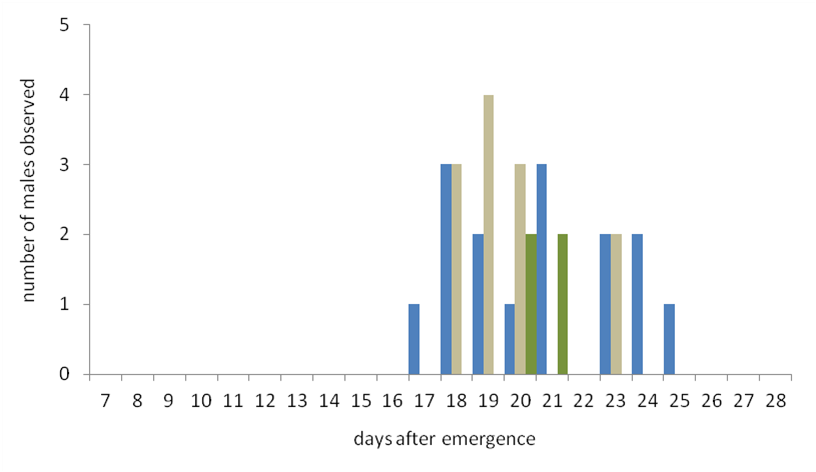

Supplement: Additional file 2: Figure S2. — Aging effect on male sperm viability (proportion of live to dead sperm cells), for males of two different colonies (each color represents a colony). Between nine and ten males per colony were analyzed weekly, from 1 week old to 5 weeks old. Median values are represented by the lines inside the boxes, which span the first and third quartiles, and points represent outliers. (TIF 91 kb) [file 12862_2016_765_MOESM2_ESM.tif]

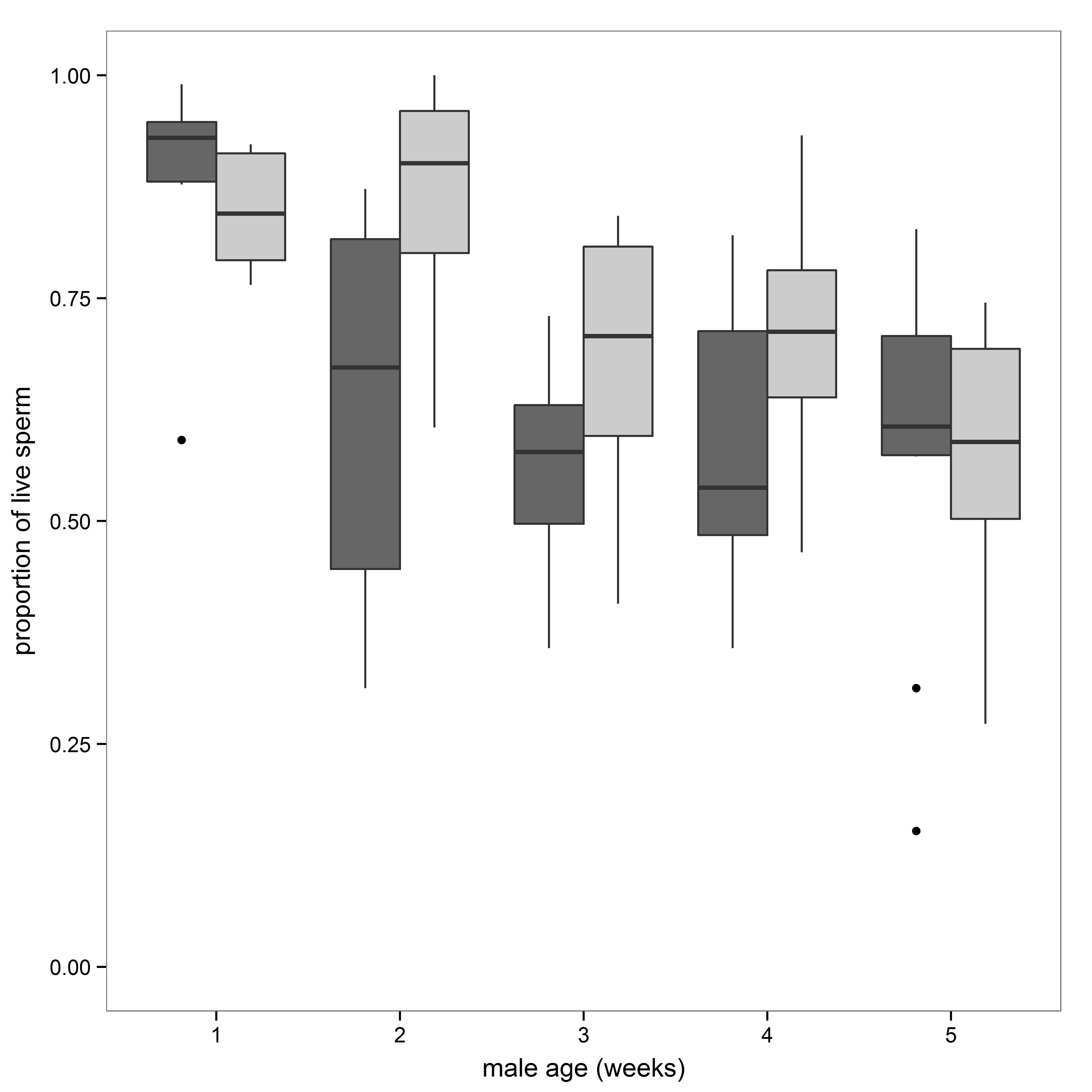

Supplement: Additional file 3: Table S1. — Male traits assessed in this study. (TIF 185 kb) [file 12862_2016_765_MOESM3_ESM.tif]

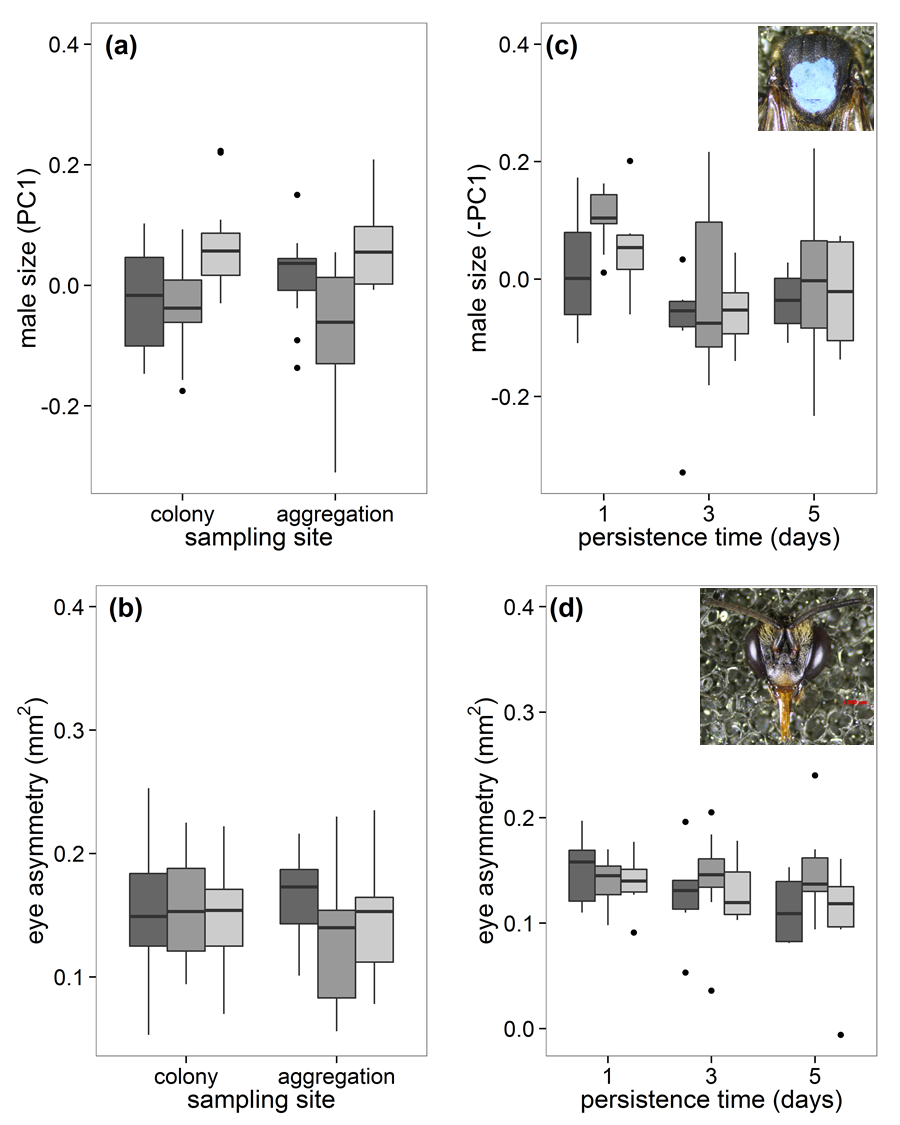

Supplement: Additional file 4: Figure S3. — Morphological traits related to male competitive ability (male’s ability to reach and persist in aggregations). Neither male body size nor eye asymmetry influenced the male’s ability to reach an aggregation (a-b, each color represents a colony). Males that persisted more days in the aggregations were smaller, but did not show different eye asymmetry (c-d). Each color represents an aggregation (one aggregation was used in 2014 and two aggregations were used in 2015). Median values are represented by the lines inside the boxes, which span the first and third quartiles, and points represent data outside 1.5 times the interquartile range above the upper quartile and bellow the lower quartile. (TIF 326 kb) [file 12862_2016_765_MOESM4_ESM.tif]

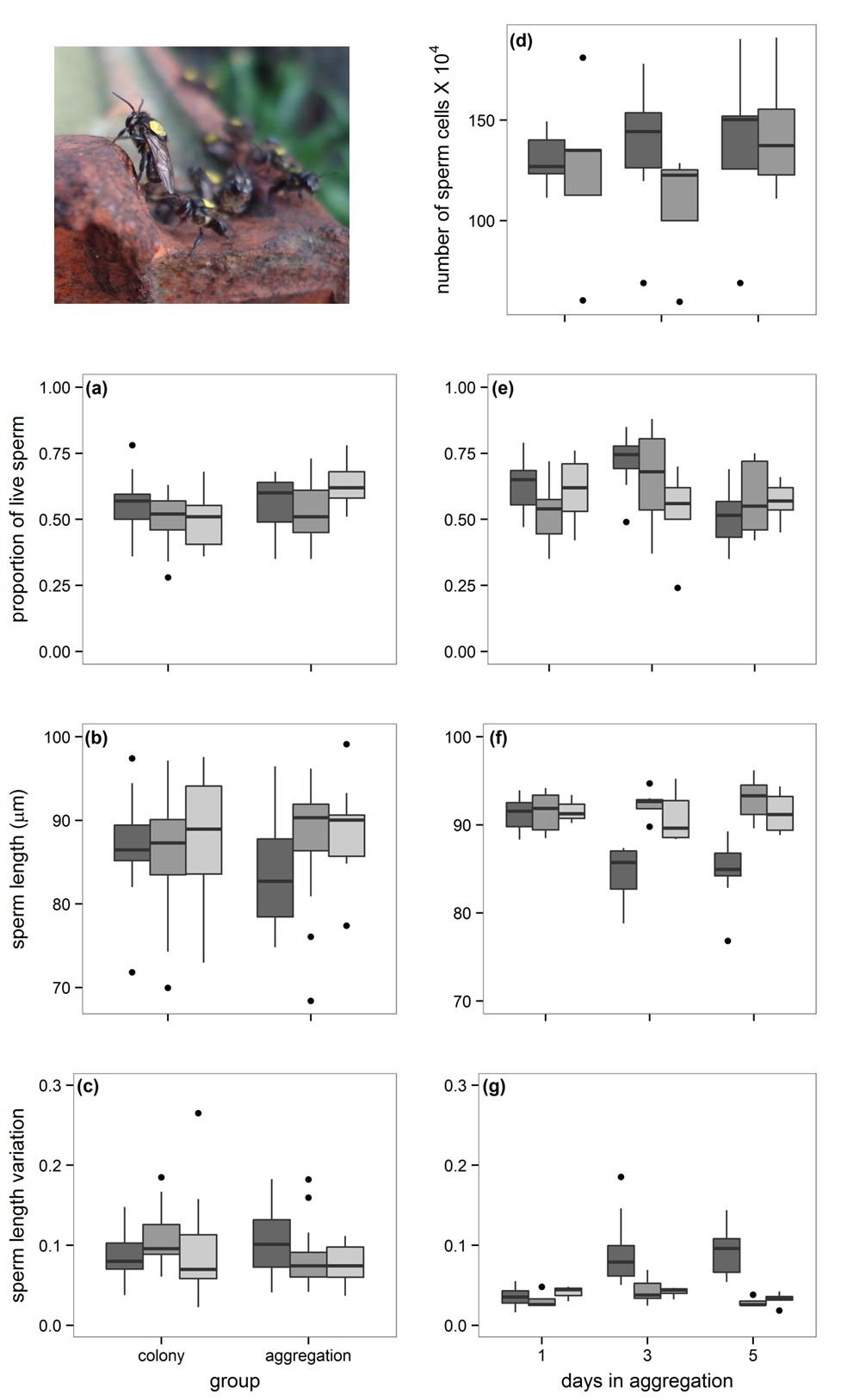

Supplement: Additional file 5 — Figure S4. (a-c) Sperm traits of males collected inside the colonies and males that reached aggregations. Each color represents a colony. (d-g) Sperm traits of males with different persistence times in the aggregations (new-coming males and males that persisted for 3 or 5 days). Each color represents an aggregation (one aggregation was used in 2014 and two aggregations were used in 2015). Median values are represented by the lines inside the boxes, which span the first and third quartiles, and points represent data outside 1.5 times the interquartile range above the upper quartile and bellow the lower quartile. (TIF 712 kb) [file 12862_2016_765_MOESM5_ESM.tif]
